# CLD4SDGs: Integrated assessment of progress and impacts on Sustainable Development Goals using Causal Loop Diagrams

**DOI:** 10.1016/j.mex.2022.101851

**Published:** 2022-09-08

**Authors:** Andrea S. Downing

**Affiliations:** Stockholm Resilience Centre at Stockholm University, 10691 Stockholm, Sweden

**Keywords:** Social-ecological systems, Trade-offs and synergies, Impacts and pathways, Leverage for change

## Abstract

The United Nations’ 2030 Agenda for sustainable development calls for meeting the global Sustainable Development Goals (SDGs) through local action and integrated approaches. We here describe a method developed to understand how local (un-)sustainable processes in coupled social-ecological systems contribute to or hinder meeting SDGs at the target-level in coupled social-ecological systems (SES). The steps include:

• The construction of a causal-loop diagram (CLD) of the social-ecological processes that shape system dynamics
• CLD simplification for the purpose of the SDG analysis,
• Steps of the SDG analysis.

The methods combine and build on published examples of CLD and SDG analyses and includes instructions for the transparent documentation of the analyses to support review and further development of SDG-target analyses in complex social-ecological systems. A template for the documentation and analysis is provided in the supplementary materials.

## Graphical abstract

**Figure:**
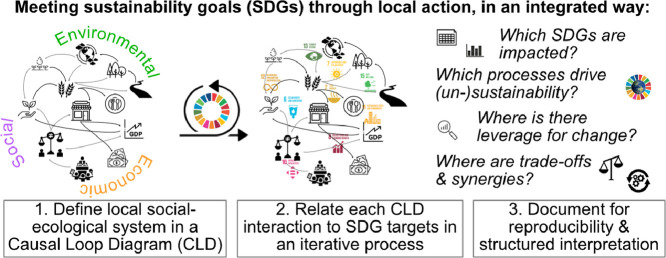

Specifications table

**Table:** 

Subject area:	
More specific subject area:	Sustainability science
Name of your method:	Causal Loop Diagrams for the integrated analysis of Sustainable Development Goal impacts and pathways: CLD4SDGs
Name and reference of original method:	Downing, A. S., G. Y. Wong, M. Dyer, A. P. Aguiar, O. Selomane, and A. Jiménez Aceituno. 2021. When the whole is less than the sum of all parts – Tracking global-level impacts of national sustainability initiatives. *Global Environmental Change* 69:102,306.
Resource availability:	*N.A.*

## Method details

The United Nations’ 2030 agenda states the Sustainable Development Goals (SDGs) as integrated and indivisible, designed at the global level and dependant on local action [1]. However, methods for integrated monitoring, that inform pragmatic and integrated implementation at local and national levels are still lacking. Existing monitoring frameworks rely on data for selected indicators, that are assessed by a ‘traffic-light’ coding system indicating how close (green) or not (red) the indicator value is to the target (e.g. [[2], [3]],). These traffic-light codes are then generally tallied or summed to get an overview of which and how many targets are being met. Such a tally over targets and goals gives a misleading impression of integration of the SDGs, when in fact it simply gives an aggregated picture of un-integrated metrics. Indeed, each indicator of sustainability can in fact be met ‘unsustainably’. As an example, the target of sustainable and just food production (target 2.3) includes an indicator for production yields (indicator 2.3.1), that is independent from but equal to the target for agricultural area under productive and sustainable agriculture (indicator 2.4.1). The presence of high yields (green point for 2.3.1) contributes to a green value for target 2.3, and is not affected by an absence or ‘red’ value on 2.4.1. This implies that high yields, irrespective of the sustainability of the processes that lead to high production, or to the distribution of this production lead to a green traffic light, without actually indicating whether the production system represents or contributes to sustainable and just food production. This is a classic case of metrics that have become goals to themselves rather than monitoring objects [4].

We present here methods that aim to understand how local (un-) sustainable practices contribute, or not, to meeting the SDGs from a systems perspective, investigating the sustainability of system processes themselves, rather than a snapshot-indicator level assessment. Our methods 1) address the SDGs in an integrated way, by mapping both the multiple goals and targets influenced by any single system process, as well as the multiple system process that shape the progress on single targets and goals; 2) provide tangible information on what kinds of initiatives can address different goals, and 3) how such initiatives can best contribute to meeting goals and limit undesired side-effects; 4) indicate which processes hinder the achievement of goals and 5) point to actors or actor groups that could play a role in making the necessary changes towards meeting the goals. These methods are designed for the SDG framework specifically, but the approach is generalizable for generating integrated sustainability pathways and scenarios.

### System representations in causal loop diagrams

The first step of the analysis consists in creating a system representation that encompasses at least the social, ecological and economic aspects of a system of interest. Though causal loop diagrams (CLDs) can technically be designed for any dynamic system, we here focus on the design of CLDs that represent social-ecological systems through all three pillars of development, to best integrate the analysis across SDGs.

**Fig. 1:**
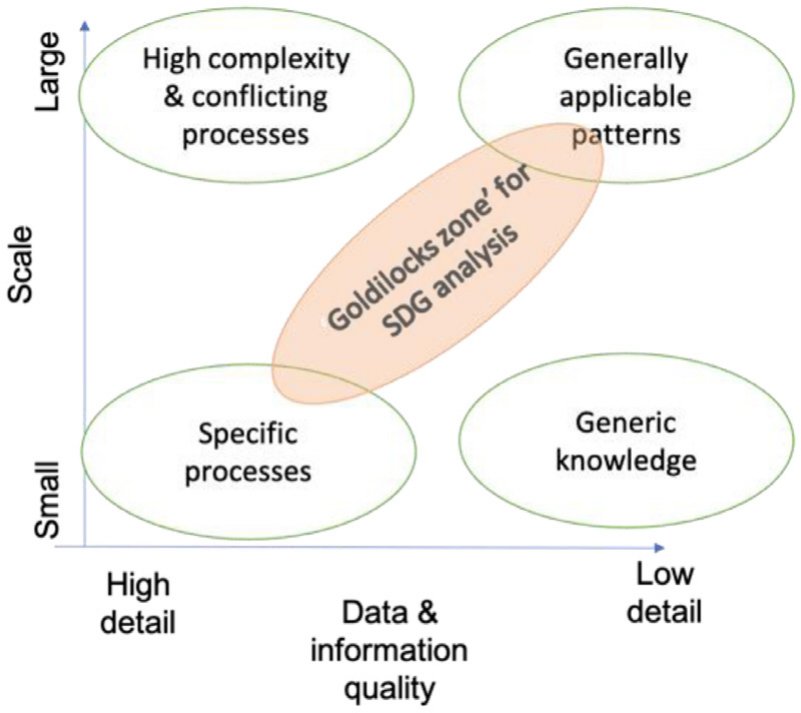
A local level of analysis for example can allow to identify specific drivers of system dynamics, and a resolution of analysis (i.e. detail level) that identifies mechanisms by which dynamics play out. A too narrow system definition leaves out many actors and driving processes, and a too broad level will lead to describing diffuse actor groups and conflicting trends. Resolution often also depends on data availability, and the scale or level of analysis should match the resolution (low resolution-larger scale; high resolution – smaller scale). Larger-scale, high resolution representations are useful for identifying macro processes, and identifying consensus at a bigger will be complex – potentially revealing multiple conflicting mechanisms driving generic dynamics in different ways (e.g. a detail of different processes that contribute to diverse weather phenomena) – and will make it challenging to clarify which processes lead to different outcomes. Small-scale, low-resolution analyses will lead to generic mechanisms for specific dynamics, and make it challenging to identify specific initiatives or actors to leverage.

The level and resolution of analysis needs to be specific enough to identify relevant actor groups, and dominant trends (Fig. 1).

### Components of a causal loop diagram

Causal Loop Diagrams (CLDs) are conceptual representations of systems, that are generally created to understand the multiple driving forces and cascading effects of a characteristic system process: for instance the connected drivers and impacts of fishing and eutrophication in Lake Victoria [5]; drivers and cascading effects of crop residue burning in the Indian State of Punjab [6]; distal and diverse cascading effects of Chinese demand for traded commodities [7]. A CLD is comprised of elements (e.g. a fish stock) connected by arrows from a source to a sink element. A positive sign on the arrow indicates that an increase in one element leads to an increase in another (e.g. when an increase in nutrients leads to an increase in fish stocks), a negative sign on the arrow indicates that an increase in an element causes a decrease in the other (e.g. increased fishing efforts leads to reduced fish stocks).

### Constructing a CLD

**Fig. 2:**
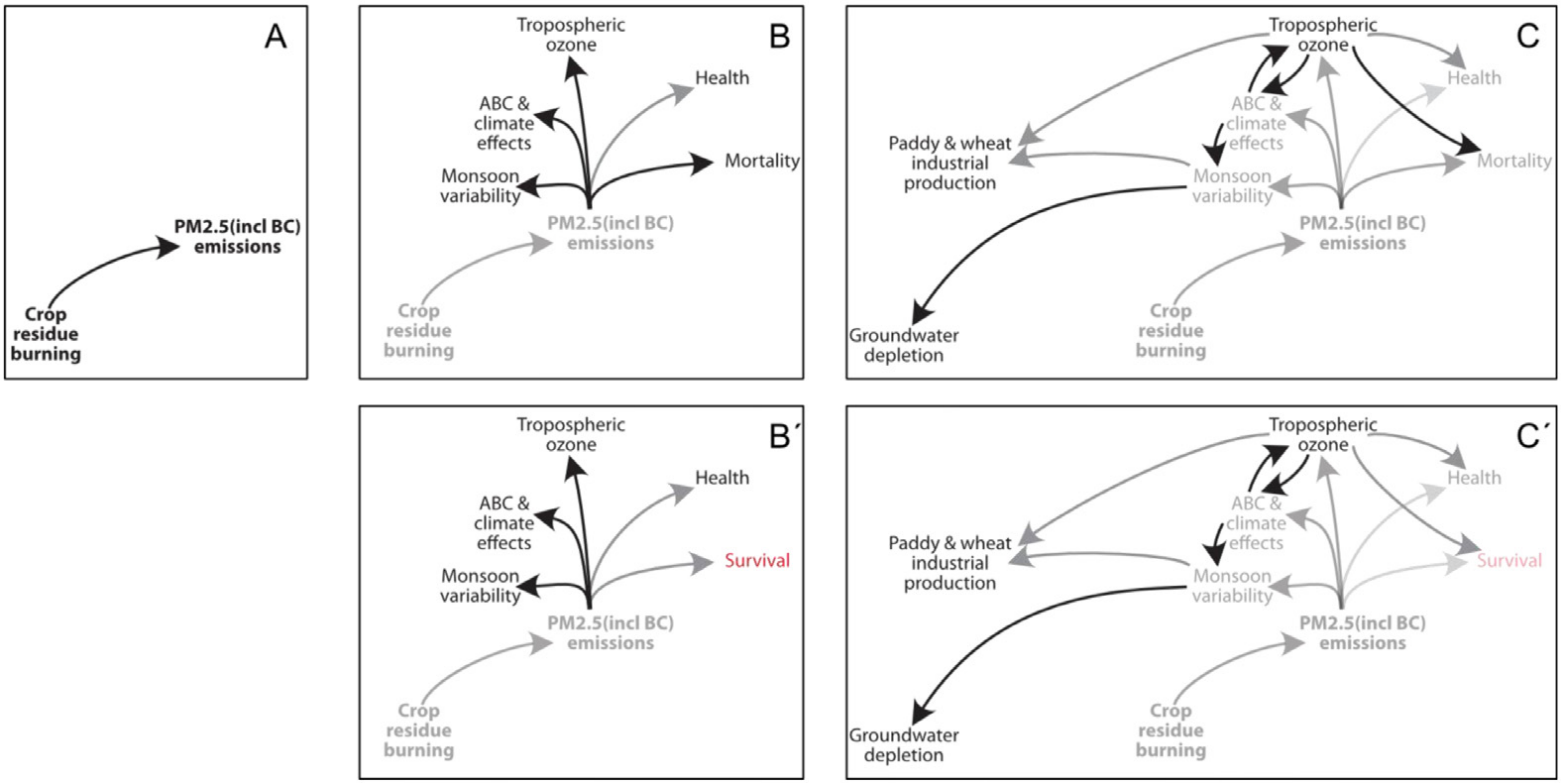
Constructing a causal loop diagram from the central element (here emissions caused by crop residue burning) in a step-wise centrifugal way (from A to C). Black arrows indicate positive interactions – where an increase in the source element leads to an increase in the receiving element – and grey arrows are negative interactions (an increase in the source leads to a decrease in the receiving element). The sign of the arrow depends on the terminology used where increased emissions can be seen to increase mortality (B), or decrease survival (B’). The choice will lead to consistent changes throughout the CLD(compare C and C´). Figure drawn from [6].

To ensure the CLD is designed to represent the key problematics of the system at hand, we recommend starting precisely at the point of the problem representation (for example crop residue burning in Downing et al., (2022)), and expanding from this point in centrifugal way (Fig. 2A, 2B, 2C). For an element at a time, finding all the interactions from drivers and to cascading effects, and then radiating out from these drivers and cascading effects each in turn.

The name of element can often be phrased in different ways, influencing the sign of the arrow – for example a positive arrow towards an element titled ‘decreased lighting’ represents the same process as a negative arrow towards an element titled ‘increased shading’ (compare Fig. 2B with 2B´, and 2C with 2C´). For consistency and simplicity, we recommend choosing first the clearest formulation of the element (or instance by avoiding double-negative formulations), and if both alternatives are equally clear, selecting then for a formulation that creates a positive arrow.

Sometimes, an interaction can be non-linear, for example where an increase in the source element at first causes an increase in the sink element, but past a threshold this relation shifts. In such cases, it is important to make note of the non-linearity, and – depending on the goals of the analysis – to either indicate both signs, or choose explicitly the interaction type of choice (pre- or post- threshold for instance). Non-linearities can also be solved by increasing the ‘resolution’ of the analysis: for instance by adding the intermediate element that creates the threshold: in Downing et al., (2014) this was represented in eutrophication being first a source of growth of lake fauna through enrichment, but then as a source of mortality through asphyxia. By producing the intermediate step of ‘anoxia and shading’, the diagram could then be used to reflect both the positive and negative effects of added nutrients, and thus remain relevant for different areas of the lake or time-frames where one or the other process was dominating. An increase in resolution is preferrable for an SDG analysis.

Creating a CLD is an iterative process with iterations consisting of a) identifying the different elements of the system and their causal connections; b) reflecting on the dynamic patterns that would play out from the feedbacks at a system level; and c) validating the connections identified by using adequate sources (literature, data, etc.). There are choices to make regarding the resolution or detail of the CLD to match the relevance for the study and availability of data. Gaps in data and knowledge that prevent higher detail can be flagged as focus areas for further research. A consolidated CLD is one where the feedbacks – sequences of interactions from one element back to itself, represent the same dynamics irrespective of the elements selected or terminology used. ‘Dead end’ interactions are best left out if they do not contribute to the understanding of system dynamics or their importance, however, if they represent important sustainable development outcomes, then they should be kept. In Downing et al., (2022) for example the elements ‘food security’ were not further connected, but kept in the CLD because they illustrate the features of future policies that need to be considered.

### Simplifying the CLD

When a coherent system representation is achieved, with an understanding of the processes that drive changes to and from social, economic and environmental spheres, the diagram can be simplified so that it maintains the same system-dynamic properties, but focuses on the elements that can directly be acted upon. For example, elements that only see positive incoming and outgoing arrows can be removed, without altering system dynamic properties such as feedbacks. This can be relevant for various analytical and communication purposes, where simpler diagrams are often more tractable. However, some such elements are actionable – such as policies or practices – and are central to describing leverage for change in the system: described in terms of where in the system, what types of changes (e.g. adding or removing interactions; adding or removing elements), and even by whom. For the purpose of an SDG analysis, we suggest first distinguishing the actionable processes from the non-actionable ones. For example, in Downing et al., (2022), the element ‘crop residue burning’ is actionable: human agency is behind whether this process takes place or not. The element ‘PM2.5 and BC emissions’ is in this context itself not actionable – if burning takes place, emissions take place. Furthermore, the ‘PM2.5′ element is only surrounded by positive arrows, it thus has no system dynamic properties. To simplify the diagram without losing the information this interaction carries, we remove non-actionable intermediate process from the diagram, connecting the (all positive) arrows that lead to it, to those that leave from it. In the table, we merge the information that related to this intermediate element to the new source-sink interactions that bypass-it. For example, in Downing 2022, we connect ‘Crop residue burning’ with ‘ABC & Climate effects’ and combine the information from the previous two interactions into one. The simplification process should thus be compiled into a separate table with the same headers as Table 1, so as to track the simplification process without losing any information.

**Table 1:** The documentation of the CLD. This documentation format enables filtering for different interaction types, source or sink elements and for the different sources or quotes that might be used multiple times in the CLD. Furthermore, this structure allows for transparency and to update the CLD with new data. Note that where multiple references or quotes exist it is worth adding them. This may be especially true for low-resolution CLDs, where an interaction representing a broad process can be captured by several references. For a concrete example of this documentation, see appendix B in supplementary materials of [6].

Arrow #	Source element	Sink element	sign	Interpretation of interaction	reference	quote	note
1	*Element from which causal arrow leaves*	*Element to which arrow points*	*(+);(-); (+/-); ?*	*More … leads to more/ less …*	*Add source of information*	*Add specific quote supporting interpretation*	*e.g. comment regarding validity of quote, or context for interaction sign
2	…	…	…	…	…	…	…

**Box 1: Checklist for the CLD**

▪ Key problem, it's causes and cascading effects are central and clearly articulated through causal interactions
▪ CLD contains elements pertaining to social; ecological and economic drivers and consequences of change
▪ ‘Dead end’ elements are justified by the problem description
▪ Causal interactions are documented and explained in a reference table
▪ Non-linear processes contextualized or handled into parallel subprocesses.
▪ Differences in wording of elements or in illustrating the causal relationships do not alter feedback signs.
▪ Key drivers are described in terms of actionable elements
▪ Non-actionable elements that are surrounded by positive arrows are removed

Alt-text: Unlabelled box

### Using the sustainable development goals framework

The UN's 2030 agenda is composed of 17 SDGs that represent social, economic and environmental facets of sustainable development. Each goal is delineated by a set of targets that to some degree integrate these facets, and provide connections across the goals: for instance, the targets of goal 2 – zero hunger – specify addressing the goals in equitable ways, and promoting agricultural diversity – thus linking equity and environmental goals [1].

There are a total of 169 targets delineating more specifically how the goals are to be met. The SDG framework also includes many more indicators designed to describe progress towards targets. Because the indicator level is country-specific, incomplete (not all goals and targets have specified and measurable indicators) [8], and that indicators in themselves do not represent sustainable outcomes, we present a method for analyses at the target level that allows both high specificity (it is relatively adaptable to different local contexts) as well as generalizable knowledge in terms of the classification of targets. Indeed, targets represent different types of aims, from changed trends, to specific outcomes or general policy directions – which implies that they can be met or contributed to by a wide variety of actors at different levels – from international & national policy, through businesses and entrepreneurs to the population at large.

The aim of the SDG analysis is to identify what the SDG target level impacts are of each actionable element, by investigating all the outgoing interactions of actionable elements.

We recommend an iterative approach to the SDG target analysis, with the following steps of iteration:

i. Search the SDG target list for terms in the descriptions and quotes of each actionable interaction. Add the targets in a column adjacent to each interaction – many targets can be identified for each interaction (Appendix A: sheet ’Target Analysis’ contains the Goal and target list – enter data in sheet ‘SimplifiedCLD_SDGimpacts’, next to each interaction).
ii. For all targets of each interaction, check the goal-level coherence, see if any other targets address or are affected by the process, add explanatory notes where necessary.
iii. For all targets of each interaction, add a sign indicating if the interaction contributes to meeting the target (+), or not (-), or it is not known (?). Note that this sign can be different to the sign of the interaction itself. For example, an interaction representing a decrease in pollution (- interaction sign) can contribute to meeting sustainable production targets (+ SDG target sign).
i. (Optional): indicate if the target is influenced by the system itself (e.g. increased food production in the system can lead to hunger reduction targets) or whether addressing the target can support changes in the system (e.g. climate policies of target 13.2 can support driving sustainable practices in the system).
v. Consolidate for each of the targets whether and how often they are addressed. This table should have each target as a row, and columns indicating a) how many interactions contribute to meeting the target; b) how many to going against the target; c) how many are unknown and d) if step iv above is taken, if the target can be used to leverage changes towards sustainability in the system. This table can be used for as system-wide analysis of which SDG targets are most impacted by the system's social-ecological interactions and how (Appendix A: complete sheet ‘Target Analysis’.

### Interpretation of results

Patterns in the analyses can reflect several factors, and we therefore suggest the following four lenses for the interpretation of results:

i. Patterns that are a product of the CLD focus. The SDG analysis cannot reveal more than was included in the CLD, and biases in the elements considered important to SES dynamics will be reflected in the SDG target pattern obtained. It is therefore relevant to scrutinize why certain targets and goals are not represented, or over-represented.
ii. Patterns that are a product of the SDG framework formulation. The SDG patterns obtained do not only reflect effective sustainability impacts, but also how dimensions of sustainability are worded in the SDG framework. For instance, few targets under Goal 13 (climate change) are specific to sources of emissions, and a CLD that represents a fairly detailed understanding of emission sources may – counterintuitively – not necessarily have many connections to SDG 13. Again scrutinizing absences of impacts, and reflecting on the sustainability of processes beyond the limitations of the SDG framework is essential in a comprehensive interpretation of results.
iii. Patterns that reflect a gap in the data. Certain CLD processes cannot be documented for lack of data and observations. Also, even where a connection can be identified between a CLD interaction and a target, the sign may not be determinable, for lack of data and monitoring. Such gaps can be useful pointers for future research.
iv. Patterns in the SDG analysis reflect documented sustainability challenges and contexts for the system described. This dimension is to some degree the validation of the analysis.

The methods above describe the decision criteria, methodological steps, validation criteria, documentation processes, and guides for interpretation of results for the integrated and systematic analysis of sustainability impacts and pathways in social-ecological systems. This approach has been used to describe the country-specific and diverse SDG impacts of agricultural commodity exports to China (see [9]), that allows to identify both the specific processes that support or go against meeting goals in each different context, as well as the more generalisable processes that can contribute to meeting goals. Built on the premise that an integrated approach depends on a systems understanding, we here provide a clear tool to build the required systems conceptualisation that can guide the analysis of sustainability impacts and pathways for change. The approach presented is versatile in that it can be designed from literature data, as well as interview or participatory workshop processes, where the key element of quality lies in the documentation of the CLD elements and interactions – which can then be built upon, deliberated and tested in a structured way, and in the impacts the CLD can be used to analyse. We here provide a specific example of the United Nation's 2030 agenda and SDG framework, but we see this approach as applicable to other sustainability or development frameworks, such as the ‘Doughnut Economics’ framework for example [10], as the social-ecological processes that shape the CLD can be assessed in an integrated way in terms of their contributions to, or against, meeting different social foundations and environmental ceilings.

## Ethics statements

Our work does not involve human subjects, animal experiments or social media data. However, should the analyses be carried out using interview or workshop data, they should then comply with the necessary and appropriate ethical and privacy requirements.

## CRediT authorship contribution statement

**Andrea S. Downing:** Conceptualization, Methodology, Validation, Writing – original draft, Visualization.
